# Birth control knowledge among freshmen of four Italian universities

**DOI:** 10.1038/s41598-020-72200-6

**Published:** 2020-10-05

**Authors:** L. Cegolon, M. Bortolotto, S. Bellizzi, A. Cegolon, G. Mastrangelo, C. Xodo

**Affiliations:** 1 Public Health Department, Local Health Unit N.2 “Marca Trevigiana”, Treviso, Italy; 2 FISPPA Department, Padua University, Padua, Italy; 3 Present Address: Medical Epidemiologist, Independent Consultant, Geneva, Switzerland; 4 Department of Political Sciences, Communication and International Relations, University of Macerata, Macerata, Italy; 5 Department of Cardiac, Thoracic & Vascular Sciences and Public Health, Padua University, Padua, Italy

**Keywords:** Medical research, Epidemiology

## Abstract

Since sexual health education (SHE) is not mandatory in Italian schools, we conducted a survey on freshmen of four Italian university campuses in 2012 to investigate the respective level of sexual health knowledge (SHK) in relation to birth control, with the aim to inform public health policy makers. A convenience strategy was employed to sample 4,552 freshmen registered with various undergraduate courses at four Italian universities: Padua university (Veneto Region); university of Milan (Lombardy Region); university of Bergamo (Lombardy Region); university of Palermo (Sicily Region). We investigated the level of SHK on birth control using 6 proxy indicators: (1) the average length of a woman’s period [outcome with 3 levels: wrong (base) vs. acceptable vs. correct]; (2) the most fertile interval within a woman’s period (binary outcome: correct vs. wrong answer); (3) the event between the end of a period and the beginning of the next cycle (binary outcome: correct vs. wrong answer); (4) the average survival of spermatozoa in the womb (binary outcome: correct vs. wrong answer); (5) the concept of contraception (binary outcome: correct vs. wrong answer); (6) the efficacy of various contraceptives to prevent unintended pregnancies (linear score: 0–17). We fitted 6 separate models of multiple regression: multinomial for outcome 1; logistic for outcomes 2, 3, 4, 6; linear for outcome 6. Statistical estimates were adjusted for a number of socio-demographic factors. Results were expressed as odds ratios (OR) for the 4 multiple logistic regression models, linear coefficients (RC) for the linear regression model and relative risk ratio (RRR) for the multinomial logistic regression model. The level of significance of each risk estimate was set at 0.05. The level of SHK of freshmen sampled was rather low, as 60% interviewees did not know the average length of a woman’s period, the average survival of spermatozoa in the womb and the concept of contraception, whilst the most fertile interval within a woman’s period was known only to 55% of interviewees. The mean score of SHK on the efficacy of various contraceptive methods was only 5 (scale 0–17). Some categories of students were consistently and significantly less knowledgeable on birth control at multivariable analysis: males; students from the university of Palermo; those with vocational secondary school education and those not in a romantic relationship at the time the survey was conducted. The results of this survey clearly call for the introduction of SHE programs in Italian schools, as already done in several European countries. School SHE should start as early as possible, ideally even before secondary school. SHE should be holistic and delivered with a multiple agency coordinated approach involving the Ministry of Health, the Ministry of Education, University and Scientific Research (MIUR), families, schools, public health departments, primary health care providers, pharmacists, media, other.

## Introduction

Sexual health (SH) is a state of physical, mental and social well-being in relation to sexuality, and essentially refers to two issues, both linked to unprotected sex: sexually transmitted infections (STIs)—including HIV—and unintended pregnancies^1,2^, both sustained by multiple partners and/or non-use of condoms^3–6^.

About 56 million abortions were estimated to be performed globally during 2010–2014^7^. Despite wide availability and free access to various contraceptive methods, which significantly contributed to reduce the global rate of unintended pregnancies from 74/1,000 during 1990–1994 to 62/1,000 in 2010–2014^7^, 40% of the total 213 million pregnancies occurring worldwide in 2012 were still unintended, of which 50% ended up with an intentional abortion^8^. The need to interrupt an unintended pregnancy is the main reason to seek an intentional abortion, which in 45% cases is estimated to be carried out unsafely, with significant risk of death and permanent damages for the mother (about 50,000 estimated annual maternal deaths)^7,9^.

Whilst it remained basically stable from 1990–1994 to 2010–2014 in low-middle income^7^, the rate of abortion in high-income countries diminished importantly during the same timescale, due to increased access to modern contraception^7^. This was particularly the case in Eastern Europe, as a result of the collapse of communism and related health policies^7^.

If on one hand pregnancies are a natural event linked to unprotected sex, when unintended (especially amongst young and adolescent individuals) they can be an obstacle to entry into the labour market, contributing to sustain the vicious cycle of social inequality^10^.

On the other hand, women in need of contraception are also at risk of STIs. According to the World Health Organization (WHO) almost 360 million new cases of curable STIs such as chlamydia and syphilis occur annually among individuals 15–49 years old globally.

It is estimated that 24% Italian women of fertility age use poorly effective contraceptives methods, coitus interruptus (withdrawal) is practiced in 18% cases and 8% women use natural or alternative anti-conceptional devices^11,12^.

The elongation of life has stretched the sexual health needs across all ages. However, SH remains of critical social importance among younger age bands. Due to a number of reasons, including poor attention of governments on sexual health education (SHE)^13,14^, young adults in fact often do not possess the necessary knowledge to correctly ascertain the health risks associated with unprotected sex^15,16^.

Despite decades of efforts in SH promotion, the age band 15–24 years is still characterized by high rates of unintended pregnancies and STI^17–20^. Understanding the sexual behavior and sexual health knowledge (SHK) of adolescents and young adults is a fundamental step to plan preventive interventions in this age group, to collect prospective public health benefits among the general population.

From a holistic perspective, SH should be promoted in the family, in the workplace and in any educational setting, including schools^21–23^. Schools have become the most important and natural place to deliver SH promotion among adolescents and young adults. SHE in communities and schools has proven effective to successfully prevent unintended pregnancies as well as STI, and in several countries it has been included in the mandatory curriculum of students^22,24^.

School SHE started in the 70s in Scandinavia, subsequently spreading also to other European countries. With the fall of communism, SHE has been introduced also in schools of Central and Eastern European countries, and at the beginning of the current millennium also to Catholic countries of Southern Europe such as France, Spain and Portugal. However, in a few Southern European countries SHE is still excluded from school curricula or it is not mandatory yet^25^.

In Italy SHE is not mandatory. Following individual and voluntary initiatives of the respective head-teachers (responsible for these programs), SHE is being conducted only in a few Italian schools, through limited programs directed at pupils of 14–19 years of age, with identical programs across all age bands. Results from previous reports on impact of school programs with mixed methodologies, including interactive techniques, have shown a low baseline knowledge combined to students’ positive approach toward school SHE courses^26^ These programs are often restricted to one technical lesson on physiology aspects of reproduction during the whole school year^13^. Whilst different school-based SHE programs have resulted in improvement on the knowledge of STI and contraception methods, this has often not correlated with high-risk sexual behaviors reduction. Specifically, a study enrolling 2,300 students aged 13–24 years attending secondary schools and universities of Tuscany Region (Italy) highlighted how most participants declared to know common contraceptive methods such as male condom and contraceptive pill. However, only half of them declared a regular use of male condom to protect from STIs^12^.

University students represent a relevant proportion of the general population of the same age, generally belong to a younger age band (18–25 years), have just become adult and are at the beginning of the first autonomous decisional steps from their family of origin. They are exposed to important risk factors, such as multiple partners and unprotected sex which entail high rate of unintended pregnancies under 25 years of age. In particular, university freshmen constitute an age category usually at the first sexual experiences and keen to get independence from their family of origin. This group offers important information on the degree of SHK delivered in secondary schools, even though there are many other possibilities in which students can receive SHK. This can include for example, direct experience, peer group, friends and sexual partners, external courses or activities, university courses or online resources^27–29^.

In view of the above, this study has been conducted on freshmen of four Italian university campuses, with the aim to investigate their respective level of SHK in relation to birth control.

## Methods

This study employed a cross sectional design to explore the SHK level in terms of birth control, using a survey conducted with an anonymous self-reported paper questionnaire administered during university lectures of academic year 2011–2012, in presence of the respective academic educators (who also provided some guidance to fill up the questionnaire). One hour (in one single day) was given to the students to complete the questionnaire. A copy of the questionnaire used in the survey can be accessed as supplementary file (S1). Approval to conduct the study was obtained from the Ethical Committee of the University of Padua and all research methods were performed in accordance with the relevant guidelines and regulations. Since all students were 18 years of age or older, parental consent was not required. Informed consent for study participation was obtained from the study subjects.

Freshmen of four Italian university campuses (Padua, Bergamo, Milan, Palermo) located in three different Italian regions were recruited by convenience sampling, on the basis of individual negotiations with the respective academic staff, as follows:

- Veneto Region: University of Padua (research coordinating centre);
- Lombardy: University of Milan and University of Bergamo;
- Sicily: University of Palermo.

Students of educational sciences, foreign language/literature, biology, chemistry, medicine, natural sciences, engineering, architecture, political sciences and economics were selected.

### Endpoints

The questionnaire collected extensive information on socio-demographic and family background of the students and 6 different outcome measures (reported below). The latter are based on 6 multiple-choice questions, each including a set of alternatives or possible answers: the best answer to the question and a number of plausible but incorrect answers.

**Outcome 1**. *Question 71. In your opinion what is the length of the cycle (in days) in a woman with normal physical conditions?* The value had to be indicated by the interviewee, who also had the opportunity to answer “*I do not know*”. Omitted answers (= 216/4,552 = 5%) have been treated as missing values and excluded from the analysis. The answers provided by the student have been categorized as follows: 28 days: “correct” answer; 25–27 days or 29–31 days: “acceptable” answer; 0–24 days or ≥ 32 days: “unsatisfactory” answer.

**Outcome 2**. *Question 72. What is the event that defines the beginning of a new menstrual cycle?* The answers were classified into a dichotomous variable as follows: 1 (correct answer): “*menses*”; 0 (wrong answer): any other option. Blanks (= 231/4,552 = 5%) were treated as missing values and excluded from analysis.

**Outcome 3.** *Question 73. What do you think is the time period of the menstrual cycle when a woman is most likely to become pregnant?* Answers were categorized as follows: 1 (correct answer): “At half month”; 0 (wrong answer): any other option. Blanks (= 188/4,552 = 4%) were treated as missing values and excluded from the analysis.

**Outcome 4**. *Question 74. How long do you think the spermatozoa can survive in the uterine environment?* Answers were grouped into a binary outcome, as follows: 1 (correct answer): “*A few days”;* 0 (wrong answer): any other option. Blanks (= 210/4,552 = 5%) were treated as missing and excluded from the analysis.

**Outcome 5.** Question 75. The term contraception signifies: Prevention of pregnancy; Prevention of conception; Prevention of the nesting of the conception in the womb; All of the above; None of the above. The answers of students were classified as follows: 1 (correct answer): “All of the above” and 0: any other option. Blanks (= 242/4,552 = 5%) were treated as missing values and excluded from the analysis.

**Outcome 6**. Question 77. Please rank the following contraceptive methods according to their efficacy in preventing pregnancy. 1 = “high”; 2 = “medium”; 3 = “low”; 4 = “I Do Not know”. The set of possible answers is shown in Table 3. A score of 1 was given to each correct answer for the efficacy of various contraceptive methods, otherwise a value of 0 was assigned to each contraceptive option. The values associated with each 17 listed contraceptive options were summed up to create a linear score ranging from 0 (no correct answer) to 17 (all answers correct). Since there were a considerable proportion of blanks (= 1,238/4,552 = 27%), missing values were treated as “I Do Not Know” and included in the analysis as such.

### Statistical analysis

Descriptive analysis reported the distribution of variables as frequencies and percentages. Proportions were compared with the respective test of comparison. The chi square for trend was employed to assess the effect of trend for explanatory factors with more than 2 levels.

We fitted 6 separate models of multiple regression: multinomial for outcome 1; logistic for outcomes 2, 3, 4, 6; linear for outcome 6. Statistical estimates were adjusted for a number of socio-demographic factors, including factors displayed in Tables 1 and 2, related to the socio-demographic profile of the interviewee and characteristics of his/her eventual current romantic relationship. Backward stepwise selection (using *p* < 0.05 as a criterion) was adopted to build up all final regression models.

**Table 1:** Distribution of variables by university campus, undergraduate course of study and sex of interviewees.

Undergraduate course	University campus											
	PADUA Mean age: 21.1 ± 1.8 years Median age = 20 years			PALERMO Mean age: 21.8 ± 2.7 years Median age = 20 years			BERGAMO Mean age: 21.3 ± 1.5 years Median age = 21 years			MILAN Mean age = 21.1 ± 1.5 years Median age = 21 years		
	Total	Males (21.1 ± 1.8)	Females (21.1 ± 1.8)	Total	Males (21.8 ± 2.8)	Females (21.8 ± 2.7)	Total	Males (21.5 ± 1.7)	Females (21.2 ± 1.4)	Total	Males (21.1 ± 1.5)	Females (21.2 ± 1.6)
Educational Sciences	489 (26.6)	21 (3.3)	468 (39.0)	651 (37.4)	182 (41.4)	467 (36.0)	151 (22.6)	14 (7.1)	137 (29.2)	300	71 (100.0)	229 (100.0)
Literature/Foreign language	0	0	0	233 (13.4)	14 (3.2)	217 (16.7)	283 (42.4)	52 (26.3)	231 (49.3)	0	0	0
Biology/medicine/chemistry/natural sciences	483 (26.2)	117 (18.3)	365 (30.4)	270 (15.5)	39 (8.9)	230 (17.8)	64 (9.6)	31 (15.7)	33 (7.0)	0	0	0
Engineering/Architecture	545 (29.6)	383 (59.8)	162 (13.5)	344 (19.7)	142 (32.3)	201 (15.5)	82 (12.3)	63 (31.8)	19 (4.1)	0	0	0
Political sciences/Economics	325 (17.6)	120 (18.7)	205 (17.1)	245 (14.1)	63 (14.3)	181 (14.0)	87 (13.0)	38 (19.2)	49 (10.5)	0	0	0
Total	1,842	1,641 (34.8)	1,200 (65.2)	1,743	44 (25.4)	1,296 (74.6)	667	469 (70.3)	198 (29.7)	300	71 (23.7)	229 (76.3)

8 missing values for sex: 1 for Padua campus, 7 for Palermo campus. Yrs = years.

Number (N), percentage (%).

**Table 2:** Distribution of variables by university campus. Number and column percentage (%).

Variables	Classes	Total	Padua	Palermo	Bergamo	Milan
			1,842	1,743	667	300
Sex (Missing: 8)	Female	3,194 (70.3)	1,200 (65.2)	1,296 (74.7)	469 (70.3)	229 (76.3)
	Male	1,350 (29.7)	641 (34.8)	440 (25.4)	198 (29.7)	71 (23.7)
Age (years) (Missing: 24)	< 21	2,125 (46.9)	955 (52.0)	871 (50.0)	194 (29.2)	105 (36.6)
	21–24	1,956 (43.2)	770 (42.0)	574 (33.0)	441 (66.3)	171 (59.6)
	25+	447 (9.9)	110 (6.0)	296 (17.0)	30 (4.5)	11 (3.8)
Nationality	Italian	4,405 (96.8)	1,753 (95.2)	1,713 (98.3)	647 (97.0)	292 (97.3)
	Non-Italian	147 (3.2)	89 (4.8)	30 (1.7)	20 (3.0)	8 (2.7)
Undergraduate course of study	Educational Sciences	1,591 (35.0)	489 (26.6)	651 (37.4)	151 (22.6)	300 (100)
	Literature/Foreign language	516 (11.3)	0	233 (13.4)	283 (42.4)	0
	Biology/medicine/chemistry/natural sciences	817 (18.0)	483 (26.2)	270 (15.5)	64 (9.6)	0
	Engineering/architecture	971 (21.3)	545 (29.6)	344 (19.7)	82 (12.3)	0
	Political sciences/Economics	657 (14.4)	325 (17.6)	245 (14.1)	87 (13.0)	0
Previous education (Missing: 84)	Scientific/classic/university degree	2,659 (59.5)	980 (53.4)	1,199 (70.8)	341 (52.5)	139 (47.8)
	Language/ socio-pedagogical/artistic	612 (13.7)	319 (17.4)	157 (9.3)	73 (11.2)	63 (21.7)
	Vocational (Technical)	1,197 (26.8)	535 (29.2)	337 (28.2)	236 (36.3)	89 (30.6)
Residence (Missing: 29)	City centre	951 (21.0)	329 (18.0)	492 28.5)	68 (10.2)	62 (20.7)
	City Outskirt	838 (18.5)	308 (16.8)	399 (23.1)	69 (10.4)	62 (20.7)
	Town > 15,000 inhabitants	768 (17.0)	306 (16.7)	313 (18.1)	94 (14.1)	55 (18.3)
	Town < 15,000 inhabitants	1,966 (43.5)	886 (48.4)	525 (30.4)	434 (65.3)	121 (40.3)
Mother’ s nationality (Missing: 251)	Italian	4,158 (96.7)	1,683 (94.6)	1,566 (98.8)	626 (97.1)	283 (96.9)
	Non-Italian	143 (3.3)	96 (5.4)	30 (1.2)	19 (3.0)	8 (3.1)
Father’s nationality (Missing: 273)	Italian	4,163 (97.3)	1,678 (95.0)	1,578 (99.9)	620 (97.0)	287 (98.0)
	Non-Italian	116 (2.7)	89 (5.0)	2 (0.1)	19 (3.0)	6 (2.0)
Mother’s age (years) (Missing: 552)	< 45	386 (9.7)	166 (9.5)	140 (10.5)	54 (8.4)	26 (9.1)
	45–49	1,294 (32.4)	575 (33.0)	410 (30.8)	223 (34.8)	86 (30.1)
	50–54	1,345 (33.6)	632 (36.3)	374 (28.1)	224 (35.0)	115 (40.2)
	55+	975 (24.4)	370 (21.2)	406 (30.5)	140 (21.8)	59 (20.6)
Father’s age (years) (Missing: 587)	< 50	795 (20.1)	390 (22.6)	231 (17.5)	119 (18.8)	55 (19.4)
	50–54	1,384 (34.9)	618 (35.8)	415 (31.4)	243 (38.5)	108 (38.0)
	55–59	1,090 (27.5)	477 (27.6)	350 (26.5)	181 (28.6)	82 (28.9)
	60+	696 (17.6)	243 (14.1)	325 (24.6)	89 (14.1)	39 (13.7)
Father’s education (Missing: 148)	University/more	791 (18.0)	287 (16.0)	380 (22.8)	58 (8.9)	66 (22.4)
	Secondary	2,097 (47.6)	883 (49.3)	778 (46.6)	301 (46.4)	135 (45.8)
	Lower	1,516 (34.4)	622 (34.7)	510 (30.6)	290 (44.7)	94 (31.9)
Mother’s education (Missing: 104)	University/more	711 (16.0)	251 (13.9)	359 (21.3)	58 (8.8)	43 (14.6)
	Secondary	2,200 (49.5)	914 (50.5)	769 (45.6)	333 (50.7)	184 (62.6)
	Lower	1,537 (34.6)	645 (35.6)	559 (33.1)	266 (40.5)	67 (22.8)
Father’s Occupation (Missing: 361)	Manager/professionals	828 (19.8)	355 (20.6)	293 (19.0)	99 (15.7)	81 (27.7)
	Technical employees	2,077 (49.6)	814 (47.2)	839 (54.3)	281 (44.6)	143 (49.0)
	Generic employees	657 (15.7)	304 (17.6)	165 (10.7)	155 (24.6)	33 (11.3)
	Other	629 (15.0)	250 (14.5)	249 (16.1)	95 (15.1)	35 (12.0)
Mother’s occupation (Missing: 306)	Manager/professionals	747 (17.6)	272 (15.5)	341 (21.8)	81 (12.7)	53 (18.1)
	Technical employees	1,455 (34.3)	701 (40.1)	400 (25.6)	225 (35.2)	129 (44.0)
	Generic employees	1,837 (43.3)	708 (40.5)	739 (47.3)	296 (46.3)	94 (32.1)
	Other	207 (4.9)	69 (3.9)	84 (5.4)	37 (5.8)	17 (5.8)
Type of family (Missing 17)	Nuclear	3,796 (83.7)	1,488 (81.1)	1,514 (87.3)	551 (82.7)	243 (81.3)
	Other	739 (16.3)	347 (18.9)	221 (12.7)	115 (17.3)	56 (18.7)
Number of siblings	0	830 (18.2)	338 (18.4)	268 (15.4)	148 (22.3)	76 (25.3)
	1	2,432 (53.5)	996 (54.1)	884 (50.7)	375 (56.4)	177 (59.0)
	2	1,099 (22.2)	388 (21.1)	482 (27.7)	110 (16.5)	29 (9.7)
	3+	279 (6.1)	120 (6.5)	109 (6.3)	32 (4.8)	18 (6.0)
Older sibling of same sex (Missing 8)	Singleton	842 (18.5)	342 (18.6)	270 (15.6)	154 (23.1)	76 (25.3)
	Female with at least on older sister	309 (6.8)	147 (8.0)	105 (6.1)	49 (7.4)	8 (2.7)
	Male with at least on older brother	812 (17.9)	276 (15.0)	377 (21.7)	111 (16.6)	48 (16.0)
	Other	2,581 (56.8)	1,076 (58.5)	984 (56.7)	353 (52.9)	168 (56.0)
Are you currently in a relationship? (Missing: 37)	Yes	2,710 (60.0)	1,042 (56.8)	1,120 (64.9)	387 (58.9)	161 (54.2)
	No	1,805 (40.0)	793 (43.2)	606 (35.1)	270 (41.1)	136 (45.8)
**Analysis restricted to responders currently in a romantic relationship**						
Are you in love with your partner? (Missing: 42)	Yes	2,378 (89.1)	902 (87.3)	997 (91.1)	344 (90.3)	135 (84.9)
	No	64 (2.4)	27 (2.6)	18 (1.6)	10 (2.6)	9 (5.7)
	Do not know	226 (8.5)	104 (10.1)	80 (7.3)	27 (7.1)	15 (9.4)
Duration of the current relationship (months) (Missing: 29)	< 3	199 (7.4)	86 (8.3)	76 (6.9)	30 (7.8)	7 (4.4)
	3–6	218 (8.1)	81 (7.8)	86 (7.8)	38 (9.9)	13 (8.2)
	7–12	312 (11.6)	130 (12.6)	113 (10.2)	48 (12.5)	21 (13.2)
	13–24	558 (20.8)	250 (24.2)	189 (17.1)	82 (21.4)	37 (23.3)
	24+	1,394 (52.0)	486 (47.1)	641 (58.0)	186 (48.4)	81 (50.9)
Place where you first met your partner (Missing: 1)	School	557 (10.6)	228 (21.9)	217 (19.4)	84 (21.7)	28 (17.4)
	Club	555 (20.5)	206 (19.8)	233 (20.8)	83 (21.5)	33 (20.5)
	Gym	87 (3.2)	27 (2.6)	47 (4.2)	9 (2.3)	4 (2.5)
	Friend’s place	664 (24.5)	241 (23.1)	304 (27.2)	75 (19.4)	44 (27.3)
	Internet	162 (6.0)	38 (3.7)	97 (8.7)	22 (5.7)	5 (3.1)
	Charity	113 (4.2)	70 (6.7)	26 (2.3)	12 (3.1)	5 (3.1)
	Other	571 (21.1)	232 (22.3)	195 (17.4)	102 (26.4)	42 (26.1)

Results were expressed as odds ratios (OR) for the 4 multiple logistic regression models, linear coefficients (RC) for the linear regression model and relative risk ratios (RRR) for the multinomial logistic regression model. In order to increase their representativeness, results of the multivariable regression analyses were weighted for the distribution by age and sex of the Italian census population of 2011.

The level of significance was set at 0.05 for all 6 multiple regression models.

## Results

All students enrolled during undergraduate classes completed and returned the questionnaire, but the final number of tools usable for the analysis slightly reduced from 4,649 to 4,552. Respondents were broken down by 4 university campuses as follows:

- Padua: 1,842 (40.4%);
- Palermo: 1,743 (38.3%);
- Bergamo: 667 (14.7%);
- Milan: 300 (6.6%).

As can be seen from Table 1 the distribution of students by age and undergraduate course of study was fairly homogeneous, although the mean age of students of the university of Palermo (21.8 years) and Bergamo (21.3 years) was slightly higher than Padua and Milan (21.1 years both). No students of literature/foreign language were recruited from the university of Padua. The vast majority of students recruited from the university of Palermo were registered for educational sciences, with a slight prevalence of male students. By contrast, students from the University of Milan were mainly enrolled in literature/foreign language courses of study (42.8%), with a preponderance of females (49.3% vs. 26.3%). Students of the university of Bergamo were only registered for educational sciences (71 males vs. 229 females).

### Socio-demographic profile

As can be seen from Table 2:

- the entire sample was composed predominantly by females (70.3% = 3,194/4,544);
- Interviews younger than 21 years were 46.9% (= 2,125/4,528);
- 43.2% (= 1,956/4,528) freshmen were 21–24 years old and 9.9% (= 447/4,528) were older than 25;
- Students of Italian nationality were 96.8% (= 4,405/4,552) out of all respondents;
- 59.5% (= 2,659/4,468) students attended an artistic, linguistic or socio-pedagogical school; 26.8% (= 1,197/4,468) came from a vocational secondary school;
- 21.0% (= 951/4,523) freshmen resided in a city-centre, 18.5% (= 838/4,523) in a city outskirt, 17.0% (= 768/4,523) in a town with more than 15,000 inhabitants, 43.5% (= 1,966/4,523) in a town with less than 15,000 inhabitants;
- 83.7% (= 3,796/4,535) came from a nuclear family;
- 18.2% (= 830/4,550) interviewees were singleton children, 53.5% (= 2.432/4,550) had one sibling; 22.2% (= 1,009/4,550) 2 siblings, 6.1% (= 279/4,550) 3 or more siblings;
- Female students with an older sister were 6.8% (= 309/4,544), male students with an older brother were 17.9% (= 812/4,544);
- Students with a foreign mother were 3.3% (= 143/4,301); whereas those with a foreign father were 2.7% (= 116/4,279).
- Most mothers (33.6% = 1,345/4,000) and fathers (34.9% = 1,384/3,965) were in the age band of 50–54 years. Mothers older than 55 were 24.4% (= 975/4,000), whereas fathers older than 60 were 17.6% (= 696/3,965);
- 18.0% (= 791/4,404) fathers and 16.0% (= 711/4,448) mothers had a postgraduate education. Fathers with an educational level limited to junior secondary school were 34.4% (= 1,516/4,404), while the respective proportion for mothers was 34.6% (= 1,537/4,448);
- the most frequent occupations of fathers were technical/office jobs or blue-collar jobs (49.6% = 2,077/4,191), 19.8% (= 828/4,191) were professionals or managers and 15.7% (= 657/4,191) non-qualified workers;
- Most mothers were unqualified workers (43.3% = 1,837/4,246), 34.3%(= 1,455/4,246) were technical/office employees or blue collars and 17.6% (= 747/4,246) professionals/managers.

### The current romantic relationship

Student in a romantic relationship were 60% (= 2,710/4,515), 65.8% (= 2,088/3,174) among females, 46.4% (= 618/1,333) among males (p < 0.001). The percentage of interviewees in romantic relationships increased with age: 56.9% (= 1,199/2,107) among interviewees 18–20 years old; 61.6% (1,195/1,941) among those 21–24 years old; 68,9% (= 305/443) in students 25 or older (p-trend < 0.001). Among those in a romantic relationship, 89.1% (= 2,378/2,668) were in love with the respective partner, 8.5% (= 226/2,668) did not know, and only 2.4% (= 64/2,668) were not in love. Romantic relationships lasted at least 24 months in 52% (= 1.394/2,681) cases, 6–12 months in 20.8% (= 558/2,681) cases and 3–6 months in 11.6% (= 312/2,681) cases.

### Sexual health knowledge

Table 3 reports the answers to the question “*Please rank the following contraceptive methods according to their efficacy in preventing pregnancy*”. Correct answers are bold marked.

- The knowledge of the efficacy of various contraceptive methods to prevent unintended pregnancies varied by university campus, being lower among freshmen of the university of Palermo, and higher for Padua and Bergamo.
- A higher proportion of correct answers was provided for sexual abstinence (80.0%) and contraceptive pill (77.4%), with slightly higher percentages among females than males for both options. The knowledge on the efficacy of emergency contraception (EC) or ‘morning after pill’ (58.4%) and coil (32.0%) was lower, with similar proportions of corect answers between males and females within the 4 university campuses.
- Only 33.4% students were aware of the intermediate contraceptive efficacy of condom, whereas 58.5% (65.3% among males; *p* < 0.001) were erroneously convinced that a condom was more efficacious, probably not considering its eventual rupture. Only 58.2% responders were aware that withdrawal had low capacity to prevent unintended pregnancies, with percentages of correct answer similar by sex of responders, although higher for the university of Padua (62.5%) and Bergamo (63.2%).
- The knowledge of the efficacy of other contraceptive methods such as ulipristal acetate (0.8%), male pill (9.7%) and contraceptive plaster (20.9%) was very low. A low proportion of correct answers to some questions corresponded to a considerably high percentage of answers “*I do not know*”.
- Female students were fairly more aware of the efficacy of all main contraceptive methods, with the exception of ulipristal acetate (1.7% among males vs. 0.5% among females; *p* < 0.001); male pill (22.0% in males vs. 16.6% among females; *p* < 0.001), contraceptive sponge (1.7% vs. 2.7%; non-significant *p* value), cervical cap (15.3% vs. 19.0%; non-significant *p* value), transparent membrane (8.4% vs. 15.0%; non-significant *p* value) and ‘morning-after pill’ (59.5 vs. 60.5%; non-significant *p* value), although the percentage of correct answers for the latter questions were rather low in both sexes.

**Table 3:** Distribution of 17 contraceptive methods by code of efficacy to prevent unintended pregnancies (High, Medium, Low, DK = Don’t know) and sex (T = Total; M = Males, F = Females).

Code	Sex																	
	T	M	F	T	M	F	T	M	F	T	M	F	T	M	F	T	M	F
	Basal temperature			Coil			Contraceptive pill			Billing’s method			Morning-after pill			Diaphragm		
High	117 (3.1)	41 (3.7)	76 (2.8)	**1,265** **(32.0)**	**299** **(25.7)**	**964** **(34.6)**	**3,144** **(77.4)**	**875** **(73.0)**	**2,266** **(79.2)**	81 (2.2)	37 (3.4)	44 (1.7)	**2,322** **(58.4)**	**693** **(59.3)**	**1,625** **(58.1)**	818 (21.0)	192 (16.1)	622 (22.6)
Medium	622 (16.3)	157 (14.2)	465 (17.2)	1,641 (41.5)	459 (39.5)	1,178 (42.3)	714 (17.6)	226 (18.9)	484 (16.9)	382 (10.2)	122 (11.1)	260 (9.9)	1,117 (28.1)	322 (27.5)	793 (28.3)	**1,423 (36.5)**	**401 (35.3)**	**1,020 (37.1)**
Low	**1,558 (40.9)**	**402 (36.3)**	**1,156 (42.8)**	321 (8.1)	115 (9.9)	206 (7.4)	72 (1.8)	31 (2.6)	41 (1.4)	**434 (11.6)**	**107 (9.7)**	**326 (12.4)**	258 (6.5)	53 (4.5)	204 (7.3)	347 (8.9)	135 (11.9)	212 (7.7)
DK	1,516 (39.8)	508 (45.9)	1,003 (37.2)	729 (18.4)	289 (24.9)	439 (15.8)	139 (3.4)	67 (5.6)	72 (2.5)	2,834 (76.0)	835 (75.8)	1,995 (76.0)	278 (7.0)	101 (8.6)	177 (6.3)	1,308 (33.6)	409 (36.0)	898 (32.6)

	Interrupted coitus^@^			Spermicide			Condom			Transparent membrane			Sexual abstinence			Contraceptive plaster		
High	233 (6.0)	94 (8.3)	137 (5.0)	235 (6.2)	108 (9.6)	126 (4.7)	2,380 (58.3)	787 (65.3)	1,589 (55.3)	156 (4.1)	56 (5.0)	98 (3.7)	**3,152 (79.9)**	**835 (72.4)**	**2,314 (83.0)**	**812 (20.9)**	**138 (12.2)**	**674 (24.5)**
Medium	586 (15.1)	167 (14.7)	419 (15.3)	845 (22.3)	327 (29.1)	516 (19.4)	**1,363 (33.4)**	**321 (26.6)**	**1,041 (36.3)**	**362 (9.6)**	**128 (11.5)**	**234 (8.8)**	136 (3.5)	51 (4.4)	85 (3.1)	1,106 (28.4)	257 (22.7)	846 (30.7)
Low	**2,265 (58.2)**	**634 (55.8)**	**1,628 (59.3)**	**800 (21.1)**	**206 (18,4)**	**593 (22.3)**	174 (4.3)	40 (3.3)	133 (4.6)	961 (25.5)	288 (25.9)	672 (25.4)	207 (5.2)	84 (7.3)	122 (4.4)	556 (14.3)	184 (16.2)	372 (13.5)
DK	806 (20.7)	242 (21.3)	562 (20.5)	1,912 (50.4)	481 (42.9)	1,429 (53.6)	167 (4.1)	58 (4.8)	109 (3.8)	2,290 (60.8)	639 (57.5)	1,647 (62.1)	452 (11.5)	184 (15.9)	266 (9.5)	1,418 (36.4)	554 (48.9)	860 (31.3)

	Male pill			Ulipristal acetate			Cervical cap			Vaginal washing			Contraceptive sponge					
High	**378 (9.7)**	**120 (10.5)**	**258 (9.4)**	**31 (0.8)**	**19 (1.7)**	**12 (0.5)**	164 (4.3)	45 (4.0)	119 (4.4)	54 (1.4)	27 (2.4)	27 (1.0)	48 (1.3)	23 (2.1)	25 (0.9)			
Medium	490 (12.6)	167 (14.7)	323 (11.8)	233 (6.1)	88 (7.9)	145 (5.4)	**493 (12.9)**	**159 (14.2)**	**334 (12.4)**	223 (5.8)	83 (7.4)	140 (5.2)	242 (6.3)	95 (8.5)	147 (5.4)			
Low	329 (8.5)	130 (11.4)	198 (7.2)	202 (5.3)	76 (6.8)	126 (4.7)	280 (7.3)	90 (8.0)	190 (7.1)	**1,526 (39.7)**	**398 (35.3)**	**1,127 (41.6)**	**564 (14.7)**	**177 (15.8)**	**387 (14.3)**			
DK	2,691 (69.2)	722 (63.4)	1,964 (71.6)	3,338 (87.8)	934 (83.6)	2,398 (89.4)	2,887 (75.5)	829 (73.8)	2,052 (76.1)	2,037 (53.1)	619 (54.9)	1,413 (52.2)	2,975 (77.7)	827 (73.7)	2,142 (79.3)			

Number of answers provided (correct answers bold marked) with column percentages (%).

^@^Withdrawal.

### Outcome 1

From Table 4 it can be seen that only 36.3% (= 1,574/4,336) interviewees knew the exact period length in physiological conditions (28 days), with overlapping percentages of correct answers among female and male interviewees. Acceptable answers were 4.1% (= 177/4,336), whereas 59.6% (= 2,585/4,336) students provided unsatisfactory answers, with higher prevalence of wrong responses among females (61.1%) than males (55.8%). Considerable variability by university campus was found in the percentages of correct responses to this question, being higher for Padua (48.8%), followed by Bergamo (34.6%), Milan (29.9%) and Palermo (24.3%), with similar distribution by sex of the interviewees within each campus.

**Table 4:** Answers to questions on sexual health.

Outcomes	Answers^@^	Total sample			University campus			
		Total	Males	Females	Padua	Palermo	Bergamo	Milan
Outcome 1 What is the length of the cycle in a woman with normal physical conditions? (M = 216)	Wrong	2,585 (59.6)	714 (55.8)	1.864 (61.1)	829 (46.4)	1,180 (72.9)	387 (60.4)	189 (65.6)
	Acceptable	177 (4.1)	102 (8.0)	75 (2.5)	86 (4.8)	46 (2.8)	32 (5.0)	13 (4.5)
	Correct	1,574 (36.3)	464 (36.3)	1,110 (36.4)	873 (48.8)	393 (24.3)	222 (34.6)	86 (29.9)
Outcome 2 What is the time period of the menstrual cycle when a woman is most likely to become pregnant? (M: 188)	Wrong	1.969 (45.1)	679 (52.9)	1.285 (41.8)	704 (39.3)	870 (52.9)	272 (42.5)	123 (42.7)
	Correct	2,395 (54.9)	605 (47.1)	1,788 (58.2)	1,086 (60.7)	776 (47.1)	368 (57.5)	165 (57.3)
Outcome 3 What is the event that defines the beginning of a new menstrual cycle? (M: 231)	Wrong	2.526 (58.5)	651 (51.2)	1,870 (61.5)	1,022 (57.4)	954 (59.3)	373 (58.2)	177 (61.3)
	Correct	1,795 (41.5)	621 (48.8)	1,172 (38.5)	759 (42.6)	656 (40.8)	268 (41.8)	112 (38.8)
Outcome 4 How long the spermatozoa can survive in the uterine environment? (M: 210)	Wrong	2,653 (61.1)	784 (60.8)	1,862 (61.1)	986 (55.0)	1,072 (66.2)	401 (62.8)	194 (66.9)
	Correct	1,689 (38.9)	505 (39.2)	1,184 (38.9)	807 (45.0)	548 (33.8)	238 (37.3)	96 (33.1)
Outcome 5 The term contraception signifies (M: 242)	Wrong	2,596 (60.2)	838 (66.3)	1,753 (57.7)	1,085 (60.7)	1,025 (63.6)	343 (54.8)	143 (50.2)
	Correct	1,714 (39.8)	426 (33.7)	1,286 (42.3)	702 (39.3)	587 (36.4)	283 (45.2)	142 (49.8)
Outcome 6 Knowledge of contraceptive methods according to their efficacy in preventing pregnancy (range: 0–17)	Mean ± SD Median (IQ)	5.0 ± 2.9 5 (3–7)	4.6 ± 2.9 5 (2–7)	5.1 ± 2.9 5 (3–7)	5.5 ± 2.7 6 (4–7)	4.1 ± 3.0 4 (2–6)	5.5 ± 2.8 6 (4–8)	5.0 ± 3.0 5 (3–7)

Number, column percentage (%), Mean score and standard deviation (SD), M = missing values (blanks).

^@^See text.

Table 5 shows the significant results of the multinomial regression analysis on the knowledge of the physiological length of a woman’s period. As can be seen there was no difference between males and females in terms of correct answer, whereas males were more likely to provide “acceptable” answers (RRR = 2.71; 95%CI 1.80; 4.07). Students from the other three university campuses provided fewer correct answers to this point, although the estimates were significant only for Bergamo (RRR = 0.53; 95%CI 0.41; 0.68) and Palermo (RRR = 0.34; 95%CI 0.28; 0.40).

**Table 5:** Multiple logistic, multinomial and linear regression analysis.

Factors	Classes	Multinomial regression RRR (95%CI)		Logistic regression OR (95%CI)				Linear regression RC (95%CI)
		Outcome 1 (Base: wrong answer) (3,924 obs.)		Outcome 2 (4,100 obs.)	Outcome 3 (3,953 obs.)	Outcome 4 (4,237 obs.)	Outcome 5 (3,987 obs.)	Outcome 6 (3,627 obs.)
		Acceptable answer*	Correct answer**					
**Panel A**								
Sex	Female	Reference	Reference	Reference	Reference		Reference	Reference
	Male	2.71 (1.80; 4.07)	0.93 (0.76; 1.13)	0.63 (0.54; 0.74)	1.45 (1.24; 1.69)		0.76 (0.64; 0.89)	− 0.53 (− 0.76; − 0.31)
University campus	Padua	Reference	Reference	Reference		Reference	Reference	Reference
	Palermo	0.48 (0.32; 0.74)	0.34 (0.28; 0.40)	0.55 (0.47; 0.64)		0.66 (0.56; 0.77)	0.78 (0.66; 0.91)	− 1.19 (− 1.43; − 0.96)
	Bergamo	1.09 (0.66; 1.81)	0.53 (0.41; 0.68)	0.87 (0.69; 1.09)		0.72 (0.57; 0.91)	1.21 (0.98; 1.51)	0.32 (0.01; 0.62)
	Milan	0.95 (0.46; 2.00)	0.87 (0.62; 1.21)	1.13 (0.84; 1.52)		0.98 (0.72; 1.34)	1.22 (0.91; 1.63)	− 0.07 (− 0.52; 0.39)
Previous education	Classic/scientific/university degree	Reference	Reference			Reference		Reference
	Artistic/language/socio-pedagogic	0.41 (0.18; 0.91)	0.85 (0.69; 1.07)			0.87 (0.71; 1.08)		− 0.08 (− 0.21; 0.36)
	Vocational (Technical)	0.80 (0.55; 1.17)	0.61 (0.51; 0.73)			0.85 (0.73; 0.99)		− 0.24 (− 0.47; − 0.01)
Undergraduate course of study	Educational sciences	0.58 (0.35; 0.96)	0.29 (0.23; 0.36)	0.68 (0.55; 0.83)	0.71 (0.59; 0.87)	0.43 (0.35; 0.52)	1.41 (1.14; 1.73)	− 0.97 (− 1.25; − 0.69)
	Literature/Foreign language	0.35 (0.15; 0.80)	0.70 (0.52; 0.94)	0.96 (0.73; 1.28)	0.68 (0.52; 0.88)	0.82 (0.63; 1.08)	1.53 (1.15; 2.02)	− 0.96 (− 1.34; − 0.57)
	Biology/medicine/chemistry/natural sciences	Reference	Reference	Reference	Reference	Reference	Reference	Reference
	Engineering/architecture	0.74 (0.45; 1.22)	0.80 (0.63; 1.00)	1.10 (0.88; 1.38)	0.83 (0.67; 1.04)	0.78 (0.63; 0.96)	1.11 (0.88; 1.39)	− 0.67 (− 0.97; − 0.37)
	Political Science/ Economics	0.40 (0.21; 0.74)	0.47 (0.37; 0.61)	0.88 (0.69; 1.12)	0.72 (0.57; 0.91)	0.70 (0.56; 0.88)	1.41 (1.11; 1.80)	− 0.54 (− 0.87; − 0.20)
Nationality	Italian	Reference	Reference			Reference		Reference
	Non-Italian	2.20 (1.08; 4.46)	0.59 (0.36; 0.96)			0.81 (0.55; 1.20)		− 1.55 (− 2.37; − 0.73)
Mother’s nationality	Italian							Reference
	Non-Italian							0.19 (− 0.59; 0.97)
Father’s nationality	Italian			Reference				
	Non-Italian			0.43 (0.27; 0.66)				
Type of Family	Nuclear							Reference
	Non-nuclear							0.24 (− 0.01; 0.49)
Older sibling of same sex	Singleton	Reference	Reference			Reference		
	Female with older sister	0.32 (0.15; 0.69)	0.99 (0.71; 1.38)			0.65 (0.48; 0.88)		
	Male with older brother	0.28 (0.12; 0.67)	0.87 (0.68; 1.13)			0.93 (0.74;1.16)		
	Other	0.93 (0.61; 1.41)	1.01 (0.82; 1.23)			0.98 (0.82; 1.17)		
Mother’s age (years)	< 45							0.36 (0.01; 0.71)
	45–49							Reference
	50–54							0.40 (0.06; 0.75)
	55+							0.57 (0.21; 0.94)
Father’s education	University/more				Reference			
	Secondary				1.25 (1.03; 1.52)			
	Lower				1.09 (0.88; 1.35)			
Father’s occupation	Manager/professionals	Reference	Reference				Reference	
	Technical employees	1.09 (0.63; 1.91)	0.68 (0.53; 0.87)				0.84 (0.67; 1.05)	
	Generical employees	0.85 (0.52; 1.38)	0.72 (0.58; 0.89)				0.98 (0.81; 1.19)	
	Other	0.68 (0.35; 1.31)	0.83 (0.64; 1.08)				1.06 (0.83; 1.35)	
Mother’s occupation	Manager/professionals				Reference			Reference
	Technical employees				0.89 (0.73; 1.10)			− 0.11 (− 0.39; 0.17)
	Generical employees				1.01 (0.83; 1.25)			0.02 (− 0.25; 0.30)
	Other				1.43 (1.02; 2.02)			0.42 (− 0.12; 0.97)
Currently in a relationship	Yes	Reference	Reference	Reference		Reference		Reference
	No	1.23 (0.88; 1.71)	0.73 (0.62; 0.85)	0.49 (0.43; 0.56)		0.73 (0.64; 0.84)		− 0.38 (− 0.57; − 0.19)

Model restricted only to interviewees in a romantic relationship. Estimated adjusted also for socio-demographic factors displayed at the bottom of the table.								
Factors	Classes	Multinomial regression RRR (95%CI)		Logistic regression OR (95%CI)				Linear regression RC (95%CI)
		Outcome 1 (Base: wrong answer) (2,377 obs.)		Outcome 2 (2,515 obs.)	Outcome 3 (2,297 obs.)	Outcome 4 (2,295 obs.)	Outcome 5 (NA)	Outcome 6 (2,288 obs.)
		Acceptable answer*	Correct Answer**					
**Panel B**								
Duration of the relationship (months)	< 3	0.75 (0.29; 1.96)	0.96 (0.67; 1.39)	0.93 (0.67; 1.28)	1.26 (0.91; 1.73)	0.94 (0.67; 1.30)		− 0.49 (− 0.99; − 0.01)
	3–6	0.41 (0.16; 1.07)	0.72 (0.53; 0.97)	0.87 (0.66; 1.15)	1.21 (0.92; 1.58)	1.01 (0.77; 1.32)		− 0.07 (− 0.45; 0.31)
	7–12	1.26 (0.72; 2.23)	1.00 (0.79; 1.27)	0.83 (0.67; 1.04)	1.30 (1.04; 1.61)	0.95 (0.76; 1.18)		− 0.21 (− 0.51; 0.08)
	13–24	0.52 (0.18; 1.50)	0.64 (0.44; 0.94)	0.70 (0.50; 0.97)	0.75 (0.53; 1.07)	0.59 (0.41; 0.85)		− 0.36 (− 0.84; 0.11)
	24+	Reference	Reference	Reference	Reference	Reference		Reference

Relative risk ratio (RRR), odds ratio (OR) and regression coefficients (RC); 95% confidence interval (95%CI). Results weighted for sex and age to make them more representative of the Italian general population (census data 2011).

In panel A:

Outcome 1: Wrong answer = 0–24 or ≥ 31 days; *Acceptable answer = 25–27 or 29–31 days.; **correct answer = 28 days.

Outcome 1 (model with 3,924 complete observations): Knowledge of the length of female cycle in physiological conditions [answer: correct, acceptable, wrong (base)];

Outcome 2 (model with 4,100 complete observations): Knowledge of the time period of the menstrual cycle when a woman is most likely to become pregnant (Yes vs. No):

Outcome 3 (model with 3,953 complete observations): Knowledge of the event defining the beginning of a new menstrual cycle (Yes vs. No);

Outcome 4 (model with 4,237 complete observations): Knowledge of the survival of spermatozoa in the uterine environment (Yes vs. No).

Outcome 5 (model with 3,987 complete observations): Knowledge of contraception concept (Yes vs. No);

Outcome 6 (model with 3,627 complete observations): Knowledge of efficacy of various contraceptive methods (score range: 0–17)

Wrong answer = 0–24 or ≥ 31 days; *Acceptable answer = 25–27 or 29–31 days; **Correct answer = 28 days.

In panel B:

Outcome 1: Wrong answer = 0–24 or ≥ 31 days; *Acceptable answer = 25–27 or 29–31 days; **correct answer = 28 days.

Outcome 1 (model with 2,377 complete observations): Knowledge of the length of female cycle in physiological conditions [answer: correct, acceptable, wrong (base)]; Model adjusted for sex of the interviewee, undergraduate university course, university campus, previous education of the interviewee, father’s occupation, duration of the current (romantic) relationship.

Outcome 2 (model with 2,515 complete observations): Knowledge of the time period of the menstrual cycle when a woman is most likely to become pregnant (Yes vs. No): Model adjusted for sex of the interviewee, university campus; undergraduate course of study; father’s education; nationality of the interviewee; duration of the current (romantic) relationship;

Outcome 3 (model with 2,297 complete observations): Knowledge of the event defining the beginning of a new menstrual cycle (Yes vs. No); Model adjusted for sex of the interviewee, undergraduate course of study, mother’s age, duration of the current (romantic) relationship;

Outcome 4 (model with 2,295 complete observations): Knowledge of the survival of spermatozoa in the uterine environment (Yes vs. No). Model adjusted for university campus, undergraduate course of study, nationality of the interviewee; number of siblings, older sibling of same sex; mother’s age, mother’s education, duration of the current (romantic) relationship;

Outcome 6 (model with 2,288 complete observations): Knowledge of efficacy of various contraceptive methods (score range: 0–17). Model adjusted for sex of the interviewee, university campus, undergraduate course of study, previous education of the interviewee, nationality of the interviewee, mother’s age, mother’s occupation, duration of the current (romantic) relationship.

Other factors associated with a lower rate of correct answers were vocational secondary school education (RRR = 0.61; 95%CI 0.51; 0.73), non-Italian nationality of students (RRR = 0.59; 95%CI 0.36; 0.96), single status of the responder (RRR = 0.73; 95%CI 0.62; 0.85) and technical (RRR = 0.68; 95%CI 0.53; 0.87) or generic (RR = 0.72; 95%CI 0.58; 0.89) occupation of the respective fathers. Conversely, students of life sciences (reference) were more knowledgeable on the exact cycle length. Students not in a romantic relationship were less likely to be knowledgeable (RRR = 0.73; 95%CI 0.62; 0.85), especially those in a relationship for 3–6 months (RRR = 0.72; 95%CI 0.53; 0.97) or 13–24 months (RRR = 0.64; 95%CI 0.44; 0.94).

In regard to acceptable answers, female students with at least one older sister (RRR = 0.32; 95%CI: 0.15; 0.69) and male students with at least one older brother (RRR = 0.28; 95%CI 0.12; 0.67) were less likely to provide “*acceptable*” answers.

### Outcome 2

As can be seen from Table 4, 54.9% (= 2,395/4,364) interviewees provided a correct answer to the question on the fertile window within a woman’s period, with lower percentage in males (47.1% = 605/1,284) than females (58.2% = 1,788/1,285). The rates of correct answer were higher for freshmen of the University of Padua (60.7%) and remarkably lower for interviewees of the university of Palermo (47.1%).

Table 5 shows only the significant results of a multivariable logistic regression model fitted to investigate the knowledge of time window of higher fertility within a woman’s period.

Males (OR 0.63; 95%CI 0.50; 0.74), freshmen of the university of Palermo (OR 0.55; 95%CI 0.47; 0.64), students of educational sciences (OR 0.68; 95%CI 0.55; 0.83), those with a non-Italian father (OR 0.43; 95%CI 0.27; 0.66) and those not in a romantic relationship (OR 0.49; 95%CI 0.43; 0.56) were less knowledgeable about the most fertile time within a female period.

Among students non-single, those in a relationship for 13–24 months were significantly less knowledgeable of the period of time when a woman is more likely to become pregnant (OR 0.70; 95%CI 0.50; 0.97).

### Outcome 3

As can be noted from Table 4 less than half students (41.5% = 1,795/4,321) answered correctly (“*menses*”) to the question on event marking the start of a new female cycle, with a percentage of correct answers higher among males (48.8% = 621/1,272) than males (38.5% = 1,172/3,042). The rates of correct answer to this question were rather homogeneous across university campuses, with slightly higher rates for Padua (42.6%) and lower for Palermo (40.8%).

As can be seen from Table 5 males (OR 1.45; 95%CI 1.24; 1.69), students of life sciences (reference), those whose fathers had a secondary school education (OR 1.25; 95%CI 1.03; 1.52) and those whose mothers had “other” forms of employment (OR 1.43; 95%CI 1.02; 2.02) had significantly higher SHK of the event defining the beginning of a new cycle. Among non-single students, those in a relationship for 7–12 months (OR 1.30; 95%CI 1.04; 1.61) had significantly higher level of knowledge of the event defining the beginning of a new cycle.

### Outcome 4

As can be seen from Table 4, only 38.9% (= 1,689/4,342) interviewees responded correctly to the question on survival of spermatozoa in uterus, with almost overlapping percentages between males (39.2% = 505/1,289) and females (38.9% = 1,184/3,046). A remarkably higher rate of correct answer to this question was provided by students of the University of Padua (45.0%) as compared with freshmen of the other 3 universities.

Students of the university of Palermo (OR 0.66; 95%CI 0.56; 0.77) and Bergamo (OR 0.72; 95%CI 0.57; 0.91), those with vocational secondary school education (OR 0.85; 95%CI 0.73; 0.99), females with at least one older sister (OR 0.65; 0.48; 0.88) and those not in a romantic relationship (OR 0.73; 95%CI 0.64; 0.84) were less likely to know the survival of spermatozoa in the womb. By contrast, student of life sciences (reference), where significantly more knowledgeable (Table 5).

Current romantic relationships lasting 13–24 months (OR 0.59; 95%CI 0.41; 0.84) were associated with significantly lower knowledge of the survival time of spermatozoa in the uterus.

### Outcome 5

As can be noted from Table 4 only 39.8% (= 1,714/4,310) interviewees had an understanding of the concept of contraception (prevention of conception, pregnancy and nesting of the conception in uterus), with higher prevalence of correct answers among females (42.3% = 1,286/3,039) than males (33.7% = 426/1,264). Higher rates of correct answers were provided by students of the university of Milan (49.8%) and Bergamo (45.2%).

At multivariable analysis (Table 5) males (OR 0.76; 95%CI 0.64; 0.89) and freshmen of Palermo (OR 0.78; 95%CI 0.66; 0.91) had a lower knowledge of contraception definition. Conversely, interviewees from educational sciences (OR 1.41; 95%CI 1.14; 1.74), language/literature (OR 1.53; 95%CI 1.15; 2.02) and political sciences/economics (OR 1.41; 95%CI 1.11; 1.80) were more knowledgeable.

### Outcome 6

As can be seen from Table 4 the average score of correct answers to question 77 was 5.0 ± 2.9, with a median of 5 (IQ range 3–7). The average score was higher among females (5.1 ± 2.9) than males (4.6 ± 2.9). As can be seen from Table 4, students of the university of Padua (5.5 ± 2.7) and Bergamo (5.5 ± 2.8) scored higher than Milan (5.0 ± 3.0) and Palermo (4.1 ± 3.0).

Table 5 shows only the significant results of the multivariable regression analysis on the knowledge of efficacy of various contraceptive methods. Males (RC = −0.53; 95%CI − 0.76; − 0.31), freshmen of Palermo (RC = − 1.19; 95%CI − 1.43; − 0.96), non-Italian nationals (RC = − 1.55; 95%CI − 2.37; − 0.73), those whose mother was 45–49 years old (reference) and those not in a romantic relationship at the time of the survey (RC = − 0.38; 95%CI − 0.57; − 0.19) and those who were in relationship for less than 3 months (RC = − 0.49; 95%CI − 0.99; − 0.01) had a significantly lower level of knowledge. By contrast, students of life sciences (reference) were more knowledgeable about the efficacy of the various contraceptive methods.

## Discussion

### Interpretation of findings in relation to other studies

SH studies in the open literature are scarce, conducted as local surveys with low representativeness and generally not employing validated tools^1,2,30–33^. The comparison with published findings on SHK is hampered by differences among the various surveys in terms of age, sex, educational level, proportion of sexually active population and timing of investigations. Another difficulty is the variation in how SHK was measured. Some studies have assessed an *objective* knowledge through a series of true-or-false items^31,34–36^; others examined a perceived or *subjective* knowledge in relation as to whether respondents thought they knew “*much*” or “*a lot*” about specific contraceptive methods^29^. Prior research has also examined the use rather than knowledge of contraceptive methods^12,25,30,31,37,38^.

The highest score of SHK expressed by our students was on sexual abstinence (80%), followed by contraceptive pill (77.3%) and ‘morning after pill’ (58.4%). Only 33% respondents considered the possibility of condom rupture, which implies a lower efficacy of condom than other contraceptives. These percentages were shown in Table 6 together with results of prior studies evaluating knowledge of these particular contraceptive methods. Table 6 also reports for each study the sample size, the age range, educational level, country of origin as well as the calendar year of data collection. It can be seen that the present is the largest survey ever conducted in the field of SHK on birth control and, on the other hand, many research teams operated independently, unbeknownst to each other, in years close to 2012, when the present survey was carried out. With a very few exceptions, studies delved into contraceptive knowledge of young people attending secondary schools and universities. The confounding effect of age and education is therefore uncertain or low.

**Table 6:** Knowledge of three main contraceptive methods and/or their efficacy and/or their mechanism of action to prevent unintended pregnancies, among male and female adolescents and young adults; present results compared with published findings.

N. of study subjects (final number analyzed)	Condom (%)			Contraceptive pill (%)			Morning-after pill (%)			Reference; notes
	T	M	F	T	M	F	T	M	F	
4,649 (4,552)	33.4^&^	26.6^&^	36.3^&^	77.3^&^	73.0^&^	79.2^&^	58.4^&^	59.3^&^	58.1^&^	Present study, conducted in 2012 on freshmen of Italian universities of Padua, Milan, Bergamo and Palermo
1,800 (1,552)	58^&^	57^&^	59^&^	46^&^	46^&^	46^&^	46*	39*	53*	^29^ Nationally representative survey conducted in 2003 on Americans aged 13–24 years
1,185 (755)		96.5^$^		92.6^£^	92.7^£^	92.3^£^	50.4^#^	42.6^#^	53.4^#^	^34^ Survey conducted by Facebook in 2014, participants of both sexes aged 15–24 years from Belgium
1,177							38.6^&^			^31^ Survey conducted in 2012 on students aged 13–16 years in 13 secondary schools of Berlin
350 (271)	21.0^@^			13.7^@^			1.1^@^			^41^ Survey conducted in 2013 on students aged 13 to 17, from Pietermaritzburg area, South Africa
133 (119)	82**	8**	83**	34^€^	23^€^	43^€^	28^§^	26^§^	30^§^	^36^ Young people aged 14 to 24 recruited from youth centers of New South Wales (Australia) in 2012

T = Total study subjects; M = Males; F = Females.

^&^Knowledge of contraceptive efficacy;

*Knowledge of something a woman can do after unprotected sex, having heard about morning after pill;

^$^Correct answer to the question: The expiration date of a condom is not important (false);

^£^Correct answer to the question: The contraceptive pill prevents unintended pregnancies (true);

^#^Correct answer to the question: When a girl takes the emergency pill, she has also to take her contraceptive pill on the same day (true);

^@^Knowledge of most reliable contraceptives;

**Combined correct answers to the following questions:

Condoms can help prevent STIs (true);

Condoms can help prevent STIs (true);

Condoms have an expiry date (true);

Oil-based lubricants are ok when using latex condoms (false).

^€^Combined correct answers to the following questions:

Side effects from the pill usually go away within a few months (true);

The combined pill makes menstruation more regular (true);

The combined pill decreases blood loss during menstruation (true);

The vaginal ring must be inserted by a doctor or nurse every month (false).

^§^Correct answer to the question: The emergency contraceptive pill only works if taken the morning after having unprotected sex (false).

According to Table 6, the knowledge of condom as an effective contraceptive method was reported by 33% respondents in the present survey, versus 58% in a US national representative survey^29^. While the present study investigated the contraceptive methods according to an objective knowledge of efficacy in preventing unintended pregnancies, assigning them a rank (high, medium, low or “do not know”), the US study used a subjective criterion, “effective” including respondents who said “very” and “somewhat effective”, even though many expressed concerns about how well condoms can protect them, with three out of five agreeing that “*condoms break a lot*”^29^. In a cluster randomized trial carried out in youth centers across New South Wales (Australia), 19 knowledge questions answered correctly, calculated as percentage, were grouped into categories of contraceptive methods. Regarding condoms, the four questions combined gave an overall 82% rate of correct answer (Table 6). However, the correct response to one of such questions was as low as 50%^36^. The three estimates are not comparable, and therefore a lower percentage of knowledge of efficacy of various contraceptives combined in the present study did not highlight an unsatisfactory SHK among Italian freshmen, rather maybe the opposite.

According to the open literature, the most known and used contraceptives are condoms and contraceptive pills^1,25,30,33,34,37–41^. Among the above investigations, the use of morning-after pills (22%) was reported only once, in a survey conducted in 2013 on Italian university students^33^. There is evidence that the perception of the efficacy of the contraceptive methods and associated health risks influence their choice. The knowledge of efficacy of morning-after pills was higher in the present than in the US sample (58% versus 46%). The latter is the percent of adolescents and young adults who knew “*there is something a woman can do after unprotected sex and heard of emergency contraceptive pills or morning-after pills*”. Among those who have heard of emergency contraceptive pills, only 47% knew how emergency contraceptive pills work. The product of 0.65 × 0.47 = 0.31, or 31%, is the fraction of best knowledge of efficacy in this US sample, and is considerably lower when compared to our estimate^29^.

The morning-after pill can prevent unintended pregnancies if correctly used within few days of unprotected sex^42–46^. However, maybe as a result of misinformation and scarce knowledge, the morning-after pill has not proven to be effective in reducing unintended pregnancies or influencing the rates of abortions^42–44^. As shown in Table 6, the knowledge of the morning-after pill remains considerably low^29,31^. Both in high and low-income countries a poor uptake of the morning after pill has been attributed also to incorrect information on its mechanism of action and the negative perception of its side effects^47–51^. In a recent Belgian survey conducted via Facebook on individuals aged 15–24 years, the concept that a girl needs to take her contraceptive pill on the same day she has also taken the morning after pill was known only to 42.6% males and 53.4% respondents^34^. There is also evidence that the purchase of morning-after pills without prescription may enhance the spread of its use^52^.

Regarding difference by sex, in the present study male students had significantly lower SHK with several different endpoints related to birth control knowledge. Likewise, a higher level of knowledge of females in several aspects of SH was confirmed in the above survey investigating 1,177 secondary school students of Berlin^31^ and by 119 young people aged 14 to 24, recruited from youth centres of New South Wales (Australia) during 2012^36^. In an American SHE intervention on male college students, the use of a condom was encouraged through promotional videos. Students who underwent the intervention in the latter study eventually had less unprotected intercourse events and a lower number of partners as compared to those receiving standard SHE. Furthermore, the proportion of condom use at last sexual intercourse was significantly higher in the intervention group^53^.

In a study by self-administered questionnaire on 2,572 students from Serbia, Romania and Hungary during 2009–2011, a significantly higher proportion of biomedical students knew the time window of maximum fertility within a female period as compared to students of other courses: 86.0% vs. 71.5% in Hungary (p = 0.02); 71.9% vs. 61.1% in Romania (*p* < 0.001) and 59.8% vs. 43.2% in Serbia (*p* < 0.001)^54^. Biomedical students had a higher knowledge of the interval of maximum fertility in a female cycle also in the present study (61.4%), followed by students of literature/foreign language (57.1%), engineering/architecture (55.9%), political sciences/economics (53.7%) and educational sciences (50.7%).

Three hundred seventy-six students attending five public secondary schools in Rome were investigated with three questionnaires evaluating, respectively, the baseline SHK, the gain in knowledge after a SHE program, and the retention of information 4–6 months later^55^. At the pretest, the question on the fertile period within the menstrual cycle was similar to our Outcome 2. The percentage of correct answers was 42.1% (32.6% in males vs. 57.2% in females) among secondary schools’ pupils of the latter study and 54.9% (52.9% in males vs. 58.2% in females) among university freshmen sampled in the present survey (Table 4). Another question (survival time of spermatozoa in the vagina), similar to our Outcome 4, achieved 17.6% (16.2% in males, 20.0% in females) correct answers in the latter study, whereas it was 38.9% (rather balanced between males and females) in the present sample (Table 4). The consistently higher percentages of correct answers in the present survey could be due to the higher age and educational level of study subjects.

### Sexual health education

According to Table 4, the wrong answers for Outcome 1, 2, 3, 4 and 5 were, respectively, 59.6%, 45.1%, 58.5% and 61.1% (Table 4). Outcome 6 was obtained as sum of 17 scores of efficacy from 17 contraceptive methods. The score for “I do not know” was below 33% in the first tertile, between 33 and 66% in the second, and ranged from 69.2 to 87.8% in the last tertile (Table 3). Moreover, we found that freshmen of the University of Palermo had consistently lower SHK awareness as compared to their colleagues from the North of the country.

Given the demonstrated link between contraception knowledge and behaviour^35^, SHE programs may be useful in addressing risky behavior in youth populations. 95% of secondary school students surveyed from the Italian Regions of Liguria and Puglia rated schools as the main setting where SHE should be delivered; however, only 9% students considered SHE received at school as adequate, 36% rated it poor and 23% completely lacking^32^. A school-based education program managed to improve the knowledge on birth control among 559 students aged 13–19 years from Northern Italy^38^. The above-mentioned school SHE program on secondary schools of Rome, based upon workshops showed improved SHK and a positive attitude toward school SHE courses at 4–6 months since the intervention^55^.

The above studies confirmed a clear need of SHE programs in Italy, as reported also by others^12,32,56^. According to the report “Sexual Education in Europe” the level of SHE delivered in Italy is inadequate^13,57^.

SHE is an essential instrument to provide the knowledge and skills necessary to ensure adolescents can take autonomous, smart and informed healthy choices on sexuality, reducing the chance of engaging in risky sexual behaviors^14,58^. There is in fact evidence that SHE translates into a reduction of risky sexual behaviors among young people, higher mastering of contraceptive methods and lower rates of unintended pregnancies^23,59–62^. In a USA survey of young people aged 15–19, no association between contraceptive methods and sexual activity in adolescents was found. However, in the latter study adolescents receiving SHE were less likely to incur unintended pregnancies than those who did not receive any SHE or those practicing sexual abstinence^63^.

The Article 11 of the European Social Chart enforces EU member states to provide counseling and infrastructures for the delivery of health promotion, and SHE is among the fundamental elements to promote health in young people. Neglecting these public health needs constitutes a violation of the European Social Chart. Nonetheless, the EU does not have executive power to propose SHE policies within individual member states. This has led to heterogeneity of SHE across the EU^13^.

The pedagogical approaches employed to deliver SHE vary remarkably by EU countries and range from traditional formal class teaching to peer-education.

Since a survey of 1,104 US medical scholars evidenced unsatisfactory preparedness to manage SHE issues, especially in the area of safety and prevention^64^, a 1 year experimental peer-to-peer program of SHE delivered by medical students to high school scholars (aged 12–13 years) was recently tested in Rhodes Island during academic year 2015–16. The latter program consisted of 12 sessions of SHE over the course of one year, delivered to small groups of 11–15 pupils by 2 volunteer medical students in presence of a school head teacher^65^. Following these educational interventions, the targeted students became significantly more knowledgeable of various aspects of SH and more propense to interact with trusted adults to discuss issues related with sexuality^65^.

School-based SHE implies support from teachers to help young people and find answers and information to possible issues. Materials used span from films, mass-media, theatre to internet, which is increasingly used for didactical purposes, especially in game-based learning^66^ or gamification^67^. Charities and non-governmental organizations are often involved in schools as integrative sources for SHE programs. These charities have a large spectrum of tasks comprising the provision of seminars, organization of peer-education campaigns, support and reception. Health professionals are seldom involved in SHE, with the exception of a few countries employing nurses (e.g. Hungary)^14^. Even if all initiatives taken so far advocate SHE among school teenagers, there are still several constraints which limit their efficacy. As highlighted by Haruna et al.^66^ these include:

- scant government emphasis on promoting SHE;
- restricted time and space in school curricula;
- inadequate teaching and learning materials;
- ineffective traditional teaching methods;
- lack of policies;
- insufficient skilled expertise;
- taboos against socio-cultural values, such as speaking in public about sex in some countries (e.g. religious obstructionism).

The International Planned Parenthood Federation, the greatest non-governmental organization operating in the field of SH, has defined the minimum quality standards for SHE, which should not focus only on sexual and reproductive aspects, birth control, STIs and associated factors^14^. By interpreting the convention of the United Nations on childhood rights^59^, the vision of the International Planned Parenthood Federation aims to provide young people with accurate information to remove myths and legends, developing a critical thought grounded upon positive stances and values^68^.

Lectures on SHE should be exhaustive, covering multiple aspects of SH, tailored to the recipients and delivered by trained professionals, who need to be ready to answer any question from the public, without prejudice or conflict of interest^13^. The topics covered during SHE lectures should be holistic, not just focused on the physical, physiological and biological elements of human reproduction but considering its ethical, moral and emotional aspects. The topics treated should include the contraceptives available to prevent unintended pregnancies and the prevention of STIs^13^. SHE should be articulated considering various dynamics influencing the sexual preferences and the emotional, physical and social impact on personal development of each individual^4^.

A summit held in 2012 at the University of Minnesota on the current state of SHE in medical schools of Canada and the United States provided recommendations for its improvement^69^. The consensus report of the latter summit emphasized that to be effective, SH curricula need to be integrated longitudinally while requiring a multidisciplinary approach and cross-sector interaction to facilitate any process of behavioral change. The evaluation strategies should be part of the overall programs, which should comprise faculty development and cooperative strategies. A summit strategy could be interesting for other countries too, as it provides elements on “*teaching future teachers to teach*” according to Rubinstein^70^.

In Italy SHE is still a controversial topic, with public opinion and institutions inclined to maintain conservative and moralist positions. In particular, in Italy SHE has always had to face the opposition of the Catholic Church and its political affiliated groups^14^. The Concordato of 1984 established that the Italian Ministry of Education should always consult the Vatican on youth educational plans^14^. As a result, there are still no laws for the development of SHE in Italy, although several proposals and compromises have been proposed over the last decades.

Some Italian schools have started experimental programs of SHE for pupils 14–19 years old, in the form of lectures held by biology professors, with contents restricted to biology. Some schools delivered minimum programs, with just one lesson during the whole academic year, identical across all age bands; some other offer larger programs. The school head teacher is responsible for the delivery of SHE at schools. Private associations and non-governmental organizations can support these programs through activities and information centres, although they are not formally involved^13,14^.

### Strength and limitations

This survey investigated SHK on birth control in a considerable sample of Italian freshmen (N = 4,552). Sampled students came from three Italian regions, belonged to four distinct university campuses and were registered with various undergraduate courses. This aspect is relevant, as we assumed that SHK varied not only by personal and family background and geographical area, but also by undergraduate and pre-university education.

Moreover, this study investigated 6 different endpoints, allowing to explore SHK of respondents in depth and controlling for the effect of a considerable number of explanatory factors. Finally, results were weighted by sex and age of the Italian census population of 2011, to maximize the representativeness of findings.

As regards limitations, the cross-sectional design does not allow the drawing of causal conclusions. Furthermore, convenience samples were adopted and variables collected were not homogeneously distributed. However, the preponderance of females interviewed, with proportions almost identical to ours, has been reported also by others, and probably reflects the current distribution of university students by sex, in Italy and elsewhere^25^.

Data of the present survey are based upon self-reported questionnaires, hence potentially affected by recall bias and reliability of the information collected, particularly for the dichotomous outcomes created, where a proportion of correct answers may have been also given by chance. However, the linear outcome we created—on the efficacy of various contraceptive methods—combined the scores of 17 different answers, hence it is a likely reliable indicator of SHK.

Our results could be an overestimation of real SHK as other young people in the population not attending university courses could be even less “educated” about sexual health.

Finally, this study reports the findings of a survey conducted in 2012. Although a number of years have elapsed, the contribution of this study to open literature is still very relevant considering SHE is still an unresolved issue in Italy and not much has changed since 2012 in terms of school SHE. Moreover, this is the largest survey in the field of birth control knowledge conducted in Italy thus far, and no further study has explored the level of SHK in a similar structured and systematic fashion. The results of this survey could be an important reference for any comparison, in the event SHE programs would be introduced in Italy in the future.

This survey was conducted in the second half of the academic year. The level of SHK of biomedical students (particularly medicine and dentistry), who scored higher than freshmen of other undergraduate courses, may have been influenced by the type of courses already attended at the time of the survey.

## Concusions

University students sampled in this survey showed a low level of SHK for all indicators concerning birth control. This clearly calls for the implementation of SHE programs prior to undergraduate studies. SHE should start as early as possible, since the age of sexual debut is progressively decreasing^30,59^. In this perspective, SHE should be integrated into school curricula as a mandatory module, as is already happening in other European countries^71^. SHE programs should be evidence based, using multi-agency approaches involving the Italian government (particularly the Ministry of Health and Ministry of Education), schools, families, primary care physicians, public health departments, local pharmacists, media, among others^36,72^. The dissemination of the findings of the present survey through social media and press would be critical to raise the awareness of the SHE problem in the general population and in the Italian government^36,73^. School-based SHE programs should employ a holistic educational approach, adapted to adolescents’ background, their level of SHK, their socio-demographic profile and their lifestyle.

Further research should be conducted in all Italian regions to assess and monitor over time the level of birth control knowledge among university as well as secondary school students.

## Supplementary information

Supplementary file
