# Supplementary material for: Birth control knowledge among freshmen of four Italian universities
Source: Sci Rep. 2020 Oct 5;10:16466. doi: 10.1038/s41598-020-72200-6 (PMC7536290; doi:10.1038/s41598-020-72200-6)
Supplement: Supplementary file 1 — Supplementary file [file 41598_2020_72200_MOESM1_ESM.docx]

**Birth Control Knowledge Among Freshmen of Four Italian Universities**

Cegolon L,^1,2^ Bortolotto M,^3^ Bellizzi S,^4^ Cegolon A,^5^ Mastrangelo G*,^6^ Xodo C*^3^

1. Local Health Unit N.2 “*Marca Trevigiana*”, Public Health Department, Treviso, Italy

2. Institute for Maternal & Child Health, IRCCS “*Burlo Garofolo*”, Trieste, Italy

3. Padua University, FISPPA Department, Padua, Italy

4. Medical epidemiologist, independent consultant, Geneva, Switzerland

5. University of Macerata, Department of Political, Social & International Relationships, Macerata, Italy

6. Padua University, Department of Cardiac, Thoracic and Vascular Sciences, Padua, Italy

**Correspondence:**

Luca Cegolon

Local Health Unit N.2 “*Marca Trevigiana*”

Public Health Department

Treviso

Italy

email: l.cegolon@gmail.com

**Padua University**

**Scientific Coordinator: Prof. Carla Xodo**

**Bergamo University Catholic University “Sacred Heart” Palermo University**

**(Milan)**

| Dear student,  We would like to thank you in advance for taking part to this research survey entitled*:” Romantic relationships and intimacy capacity: for a sexual and emotional education of the adolescent*”, designed, funded and organized by the University of Padua, and involving also the Universities of Milan, Bergamo and Palermo.  The following questionnaire is rigorously **ANONYMOUS**. |
| --- |

**1. Your gender □ M □ F**

**2. Your date of birth (dd/mm/yyyy)**

**3. Nationality : Italian Other (specify):………………..**

**4. Your residence:**

City centre

city outskirt

town (> 15.000 inhabitants)

little town (<15.000 inhabitants)

**5. Your degree (Type of secondary school):**

**6. Number of members of your household ….**

**7. What kind of family is yours?**

- “nuclear” family (parents and children);

- naturally enlarged family (uncles, grandparents, etc besides your parents);

- family enlarged by law (please specify):

□ new partner for your father

□ new partner for your mother

□ children in law

□ step children

□ adoptive children

- Single parent household

**8. In the case of a single parent household, please indicate the condition of your parent:**

□ Single mother family

□ Widow

□ Separated

□ Divorced

□ Other (specifiy)

**9. Please indicate Nationality and date of birth of your parents:**

| **Parent** | **Year of birth** | **Nationality** |
| --- | --- | --- |
| **Father** |  | □ **Italian** □ **Other (specify)** |
| **Mother** |  | □ **Italian** □ **Other (specify)** |

**10. Indicate the educational level of your parents:**

□ Elementary school

□ Junior secondary school

□ Politechnic school

□ Secondary School

□ Undergraduate degree

□ MSc, PhD

**11. Please indicate your parents’ occupation:**

| **PARENT** | **PROFESSION** |
| --- | --- |
| **Father** |  |
| **Mother** |  |

**12. If you have siblings please fill the following table:**

| **Sibling (brother or sister)**  **from the younger on** | **Gender** | **Year of birth** |
| --- | --- | --- |
| **1°** |  |  |
| **2°** |  |  |
| **3°** |  |  |
| **4°** |  |  |
| **5°** |  |  |
| **6°** |  |  |
| **7°** |  |  |
| **8°** |  |  |
| **9°** |  |  |
| **10°** |  |  |

**13: are you currently in a relationship?**

□ Yes

□ No (please go to questions 22)

**14. If you are in a current relationship, when did it start:**

□ < 3 months ago

□ 3-6 months ago

□ 6-12 months ago

□ 12-24 months ago

□ > 24 months ago

**15. Where did you meet your partner:**

□ at school

□ in a club

□ at the gym

□ at friends’ house

□ in internet

□ in a charity event/ activity

□ Other …

**16. Are in love with your current partner?**

□ Yes

□ No

□ I don’t know

**17. When you are with your partner how often do you do these activities together? Please tick the appropriate box**

| **ACTIVITIES** | **FREQUENTLY** | **SOMETIMES** | **NEVER** |
| --- | --- | --- | --- |
| Stay home when the parents are away |  |  |  |
| Cinema |  |  |  |
| Go for a stroll |  |  |  |
| Read a book together |  |  |  |
| Go to church |  |  |  |
| Listen to music |  |  |  |
| Shopping |  |  |  |
| clubbing /dancing |  |  |  |
| Travel |  |  |  |
| Sport |  |  |  |
| Charity / voluntary work |  |  |  |
| Eat or drink together |  |  |  |
| Report and discuss what happened in our life |  |  |  |
| Sex |  |  |  |
| Watch TV |  |  |  |
| Take pictures / filming video of you |  |  |  |
| Video games |  |  |  |

**18. When you are not together ho do you communicate your presence to your partner using the following means?**

| **ACTIVITY** | **FREQUENTLY** | **SOMETIMES** | **NEVER** |
| --- | --- | --- | --- |
| SMS (texts) |  |  |  |
| MMS |  |  |  |
| Telephone |  |  |  |
| Make a surprise |  |  |  |
| Email |  |  |  |
| Write rhymes or letters |  |  |  |
| Meet in web |  |  |  |

**19. Do you argue with your partner sometimes?**

□ Frequently

□ Sometimes

□ Never (go to question 22)

**20. What are the most frequent reasons of your arguments?**

□ Jealousy

□ life approach in general

□ sexuality

□ Activity to do together

□ relationship with the respective parents

□ Lack of liberty

□ Disagreement on certain social or moral values

□ Other (specify)

**21. How do you normally resolve your arguments?**

□ we discuss and make peace soon

□ we do not see each other for some days

□ we speak by phone after the argument

□ I take the initiative(to make peace)

□ I wait for the other to get the initiative

□ Other (specify)

**22. Have you been n in a relationship in the past?**

**□ Yes □ No (please go to question 25)**

**23. How long was your previous relationship?**

□ < 3 months

□ 3-6 months

□ 6-12 months

□ 12-24 months

□ >24 months

**24. Were you in love with your ex?**

□ Yes □ No □ I do not know

**25.If you are not currently involved in any relationship , what do you think is the reason for this? Express your degree of agreement or disagreement with these statements? (Please tick each statement)**

|  | **I AGREE** | **I DO NOT AGREE** | **I DO NOT KNOW** |
| --- | --- | --- | --- |
| **I prefer to be alone** |  |  |  |
| **I do not think to be particularly attractive (physically)** |  |  |  |
| **I have a difficult character** |  |  |  |
| **I fear to suffer** |  |  |  |
| **I do not think to be ready for a relationship** |  |  |  |
| **I need to be free and independent** |  |  |  |
| **There is currently no one able to stimulate my interest** |  |  |  |
| **I have no time to dedicate to someone else** |  |  |  |
| **The person I fancy do not correspond my attention** |  |  |  |
| **I do not think to be enough interesting / charming** |  |  |  |
| **I fear to be rejected** |  |  |  |
| **It is difficult for me to take the initiative** |  |  |  |

**26. Have you ever been left by someone?**

□ Yes □ No

**27. Have you ever left someone?**

□ Yes □ No

**28. Outline FOUR (4) of the following that can be appropriate adjectives to describe a woman’s behavior in relation to emotional relationships:**

Easy Complicated Instinctive Narcissistic Affectionate De-touched

Egocentric Altruistic Reflective Optimist Jealous

Trusty Spontaneous Controlled Deep Rational

Passionate Pessimistic Generous Ambitious Selfish

Passive Disinhibited Cold Superficial Inhibited

**29. Outline FOUR (4) of the following that can be appropriate adjectives to describe a man’s behavior in relation to emotional relationships:**

Easy Complicated Instinctive Narcissistic Affectionate De-touched

Egocentric Altruistic Reflective Optimist Jealous

Trusty Spontaneous Controlled Deep Rational

Passionate Pessimistic Generous Ambitious Selfish

Passive Disinhibited Cold Superficial Inhibited

**30. When you are sexually/interested interested in someone and wish to approach hime/her you would:**

□ Take the initiative

□ try to understand if the other person could be interested

□ Wait for the other person to show some interest on me

□ Just leave it

**31. In the emotional and sexual approach towards a person you fancy, you feel (please tick max 2 options):**

□ timid

□ awkward

□ relaxed/self-confident

□ need some support from friends

□ enterprising

□ not confident

□ fearful

**32. Do you remember your “first kiss” ?**

□ yes □ No (go to question 37) □ It has not happened to me yet (go to question 37)

**33. How old were you when you first had sex?**

□ <13 years □ 14-16 years □ 17+ years

**34. Where did you have sex for the first time?**

□ at home

□ at school

□ outdoor (park, beach, mountain, etc.)

□ in a public setting (cinema, club, pub, etc

□ in a car

□ in a public transport mean

**35. What pushed you to this experience (please choose only two options)?**

□ Physical attraction

□ Emotion towards the other person

□ Desire to experience

□ Desire of emotions

□ Desire to feel adult

□ it happened without a particular reason

□ Desire to emulate my peers

**36. What emotions did you feel after this experience (please choose only two options)?**

□ Joy □ Sadness □ Pleasure □ Sense of confidence □ Indifference

□ disappointment □ Fear □ Shame □ revulsion □ Surprise □ Serenity

□ do not remember □ Felt guilty

**37. Who did you tell about your first sexual experience?**

□ Priest □ Teacher □ Friends □ Nobody □ Mother □ Father

□ Both parents □ Best friend □ A relative (uncle, Gran-parent, cousin, etc.)

**38. In your opinion how intense are the following emotions in a relationship?**

**(1=”NOT AT ALL”; 2= “A Bit” ; 3= “FAIRLY” ; 4= “VERY MUCH”)**

| **EMOTIONS** | **1** | **2** | **3** | **4** |
| --- | --- | --- | --- | --- |
| **To feel a an extraordinary energy** | □ | □ | □ | □ |
| **Appreciating ourselves more good looking** | □ | □ | □ | □ |
| **To feel closer to God** | □ | □ | □ | □ |
| **To mesmerize in front of nature’s beauty** | □ | □ | □ | □ |
| **To feel one thing with the partner** | □ | □ | □ | □ |
| **To fear loneliness** | □ | □ | □ | □ |
| **To feel generous and supportive to the other people** | □ | □ | □ | □ |
| **Not being able to concentrate** | □ | □ | □ | □ |
| **To have the feeling time spent together flies** | □ | □ | □ | □ |
| **Desire the physical union with your partner** | □ | □ | □ | □ |
| **Perceive the magic of certain places you have been with your partner** | □ | □ | □ | □ |
| **To be touched by a particular music piece** | □ | □ | □ | □ |
| **Being irritable because of defects of your partner** | □ | □ | □ | □ |
| **Desire to improve your physical appearance** | □ | □ | □ | □ |
| **Being bored when you are together with your partner** | □ | □ | □ | □ |
| **Thinking it would be too beautiful to long last** | □ | □ | □ | □ |
| **Feeling jealous** | □ | □ | □ | □ |
| **Desire to set up a family together** | □ | □ | □ | □ |
| **Fear to lose your own habits** | □ | □ | □ | □ |

**39. Have you experienced a incomplete sexual experience?**

□ Yes □ No (please go to question 44)

**40. Where did you have this first incomplete sexual experience?**

□ at home

□ at school

□ outdoor (park, beach, mountain, etc.)

□ in a public setting (cinema, club, pub, etc

□ in a car

□ in a public transport mean

**41. What pushed you to this (incomplete sexual) experience?**

□ Physical attraction

□ Emotion towards the other person

□ Desire to experience

□ Desire of emotions

□ Desire to feel adult

□ it happened without a particular reason

□ Desire to emulate my peers

**42. What emotions did you feel after this (incomplete sexual) experience?**

□ Joy □ Sadness □ Pleasure □ Sense of confidence □ Indifference

□ disappointment □ Fear □ Shame □ revulsion □ Surprise □ Serenity

□ do not remember □ Felt guilty

**43. Who did you tell about your first incomplete sexual experience?**

□ Priest □ Teacher □ Friends □ Nobody □ Mother □ Father

□ Both parents □ Best friend □ A relative (uncle, Gran-parent, cousin,etc.)

**44. Here are some youngsters’ behaviors . Please indicate whether you approve or not (in the table)**

**1= “Approve” 2= “Do not approve” 3=”I am not sure I would approve”**

| **BEHAVIOURS** | **1** | **2** | **3** |
| --- | --- | --- | --- |
| **Remain virgin until marriage** | **□** | **□** | **□** |
| **Drink alcohol beverages to feel more sociable and funny** | **□** | **□** | **□** |
| **Have sex with someone just known** | **□** | **□** | **□** |
| **Intake medicine to increase your sexual performance** | **□** | **□** | **□** |
| **Being paid to sell images of your own body** | **□** | **□** | **□** |
| **Have un-protected sex** | **□** | **□** | **□** |
| **Interrupt an eventual unwanted pregnancy** | **□** | **□** | **□** |
| **Have sex for economic or career advantages in return** | **□** | **□** | **□** |
| **Have sex only if there are underlying love feelings** | **□** | **□** | **□** |
| **Share pictures of your naked body with your partner** | **□** | **□** | **□** |
| **Recourse to the “morning after” pill** | **□** | **□** | **□** |
| **Being faithful to your partner** | **□** | **□** | **□** |
| **Have homosexual relationships out of curiosity** | **□** | **□** | **□** |
| **Having affairs without thinking too much about the consequences** | **□** | **□** | **□** |
| **Pay to have sex** | **□** | **□** | **□** |
| **Use a false profile on a social network** | **□** | **□** | **□** |
| **Masturbation** | **□** | **□** | **□** |
| **Have a virtual sexual relationship** | **□** | **□** | **□** |
| **Having cosmetic surgery** | **□** | **□** | **□** |
| **Smoking hashish or marijuana** | **□** | **□** | **□** |
| **Having sex with a trans** | **□** | **□** | **□** |
| **Having multiple simultaneous relationships** | **□** | **□** | **□** |
| **Being aggressive to impose or be considered** | **□** | **□** | **□** |

**45. you would consider your parents a couple:**

□ in love □ United □ strong □ knowing □ interacting □ in conflict

□ indifferent (to each other) □ Fragile □ Divided

**46. Have your parents ever told you about their love-story?** □Yes □No

**47. Have you ever seen your parents exchanging love gestures (kisses, hugs, caresses)?**

□ Yes □ No (please go to question 49)

**48. What do you feel when you see your parents exchanging love gestures?**

□ pleasure □ embarrassment □ indifference □ amusement

□ discomfort □ tenderness □ Other (specify)

**49. Regarding your childhood how much affection you think you have received in terms of gestures?**

□ Not at all □ A bit □ Fairly □ Very much

**50. How intensely these persons have shown their affection towards you?**

**1=”not at all” 2=”a bit” 3=”fairly” 4=” very much”**

| **FIGURES** | **1** | **2** | **3** | **4** |
| --- | --- | --- | --- | --- |
| **Mother** | □ | □ | □ | □ |
| **Father** | □ | □ | □ | □ |
| **Siblings** | □ | □ | □ | □ |
| **Gran-parents** | □ | □ | □ | □ |
| **Other relatives** | □ | □ | □ | □ |

**51. Rank the importance you give to the following ways to express your affection towards another person. 1=”not at all” 2=”a bit” 3=”fairly” 4=” very much”**

| **FIGURES** | **1** | **2** | **3** | **4** |
| --- | --- | --- | --- | --- |
| **Words** | □ | □ | □ | □ |
| **Gestures** | □ | □ | □ | □ |
| **Listening** | □ | □ | □ | □ |
| **Giving . Presents** | □ | □ | □ | □ |

**52. Have you had a complete sexual intercourse?**

□ Yes □ No (please go to question 58)

**53. How old were you (when you had a complete sexual intercourse)?**

□ < 13 years □ 14-16 years □ 17+ years

**54. Where did (your first complete sexual intercourse) happen?**

□ at Home

□ at School

□ Outdoor (garden, park, beach, mountain, etc.)

□ In a car

□ In a public transport mean

**55. What pushed you to this experience (please choose only two options)?**

□ Physical attraction

□ Emotion towards the other person

□ Desire to experience

□ Desire of emotions

□ Desire to feel adult

□ it happened without a particular reason

□ Desire to emulate my peers

**56. What emotions did you feel after this experience (please choose only two options)?**

□ Joy □ Sadness □ Pleasure □ Sense of confidence □ Indifference

□ Disappointment □ Fear □ Shame □ Revulsion □ Surprise □ Serenity

□ Do not remember □ Felt guilty

**57. Who did you tell about your first sexual experience?**

□ Priest □ Teacher □ Friends □ Nobody □ Mother □ Father

□ Both parents □ Best friend □ A relative (uncle, Gran-parent, cousin, etc.)

**58. Indicate how much embarrassment you would have in each of these situations**

**1=”not at all” 2=”a bit” 3=”fairly” 4=” very much”**

| **SITUATIONS** | **1** | **2** | **3** | **4** |
| --- | --- | --- | --- | --- |
| **Appear naked in front of your parents** | □ | □ | □ | □ |
| **To be stared at by someone** | □ | □ | □ | □ |
| **Show your intimate body parts to a doctor** | □ | □ | □ | □ |
| **Be constrained to express your opinion** | □ | □ | □ | □ |
| **Listen at the door and being discovered** | □ | □ | □ | □ |
| **Be derided because of your physical appearance** | □ | □ | □ | □ |
| **Feel sick in a public place** | □ | □ | □ | □ |
| **Be the only one in your class to fail an exam** | □ | □ | □ | □ |
| **Show your internet connections to somebody** | □ | □ | □ | □ |
| **Cry in front of people you are not familiar with** | □ | □ | □ | □ |
| **Receive attentions because of your physical appearance** | □ | □ | □ | □ |
| **Read someone diary without permission** | □ | □ | □ | □ |
| **Steal from a shop** | □ | □ | □ | □ |
| **See your parents naked** | □ | □ | □ | □ |
| **Be in a party where you do not know anyone** | □ | □ | □ | □ |
| **Receive advances by somebody** | □ | □ | □ | □ |
| **Speak badly of a person that would subsequently know about it** | □ | □ | □ | □ |
| **Be negatively judged by a teacher in front of the class** | □ | □ | □ | □ |
| **Be derided for your dressing style** | □ | □ | □ | □ |
| **Be seen drunk by people that would never imagine you ever would be** | □ | □ | □ | □ |
| **Find a private photo of you in internet** | □ | □ | □ | □ |
| **Tell lie and being discovered** | □ | □ | □ | □ |

**59. Are you registered with a social network?**

□ Yes □ No (please go to question 62)

**60. Have you ever experienced an “internet relationship”?** □ Yes □ No

**61. If you answered “yes” above, did you manage to meet your partner in reality?**  □ Yes □ No

**62. Do you (currently or in the past) binge drink ?** □ Yes □ No

**63.** **Do you use (currently or in the past) drug?** □ Yes □ No

**64. How would you rate your knowledge about sexuality?**

□ Excellent □ Good □ Sufficient □ Unsatisfactory

**65. Who taught you about sexuality in the first place?**

□ Mother

□ Father

□ Both parents

□ Other relatives

□ School teacher

□ Priest

□ Sibling (brother/sister)

□ Friend

□ Specialists (physicians/psychologists, etc.)

□ Books/journals/magazines

□ Internet

□ Nobody/Nothing

□ Other (specify) ………

**66. Indicate how much sexuality in your family is:**

**1=”not at all” 2=”a bit” 3=”fairly” 4=” very much”**

| **OPTIONS** | **1** | **2** | **3** | **4** |
| --- | --- | --- | --- | --- |
| **Discussed** | □ | □ | □ | □ |
| **Revealed** | □ | □ | □ | □ |
| **Intrusively controlled** | □ | □ | □ | □ |

**67. Do you think you had sexual intercourses dangerous for your health?**

□ Yes □ No □ Do not know

**68. Do you think you had sexual intercourses dangerous for somebody else’s health?**

□ Yes □ No □ Do not know

**69. Have you ever contracted a sexually transmitted disease?**

□ Yes □ No (please go to question 71) □ Do not know (please go to question 71)

**70. If you answered “yes” above, what have you done? (please choose max three options)**

□ I asked advice to an expert friend

□ I referred to a gynecology consultant

□ I tried to resolve it on my own

□ I went to my GP

□ I went to A&E

□ I spoke with somebody of my family

□ I have spoken with my partner

**71. In your opinion what is the length of the cycle in a woman with normal physical conditions?**

Days …………. □ Do not know

**72. What is the event that defines the beginning of a new menstrual cycle?**

□ Menses

□ Ovulation

□ Conception

□ Formation of corpus luteum

□ Do not know

**73. What do you think is the time period of the menstrual cycle when a woman is most likely to become pregnant?**

□ During menses

□ Immediately after menses

□ At half month

□ Immediately before menses

□ Other (please specify) ……..

□ I do not know

□ No answer

**74. How long do you think the spermatozoon can survive in the uterine environment?**

□ < 1 day

□ A few days 24-72 h

□ 1 week

□ > 1 week

□ I do not know

**75. The term contraception signifies:**

□ Prevention of pregnancy

□ Prevention of conception

□ Prevention of the nesting of the conception in the womb

□ All of the above

□ None of the above

**76. Which of the following are the most efficacious methods to prevent sexually transmitted diseases?**

| **METHODS** |  |
| --- | --- |
| **Basal temperature thermometer** | □ |
| **Coil** | □ |
| **Anti-contraceptive Pill** | □ |
| **Billings method** | □ |
| **Morning after pill** | □ |
| **Diaphragm** | □ |
| **Interrupted coitus** | □ |
| **Spermicide** | □ |
| **Condom** | □ |
| **Transparent membrane** | □ |
| **Sexual abstinence** | □ |
| **Contraceptive plaster** | □ |
| **Male pill** | □ |
| **Ulipristal acetate** | □ |
| **Cervical cap** | □ |
| **Vaginal washing** | □ |
| **Contraceptive sponge** | □ |

**77. Please rank the following contraceptive methods according to their efficacy in preventing pregnancy. 1= “high”; 2=”medium”; 3=”low”; 4=”Do Not know”**

| **METHODS** | **High** | **Medium** | **Low** | **Do not know** |
| --- | --- | --- | --- | --- |
| **Basal temperature thermometer** | □ |  | X |  |
| **Coil** | X |  |  |  |
| **Anti-contraceptive Pill** | X |  |  |  |
| **Bilings method** | □ |  | X |  |
| **Morning after pill** | X |  |  |  |
| **Diaphragm** | □ | X |  |  |
| **Interrupted coitus** | □ |  | X |  |
| **Spermicide** | □ |  | X |  |
| **Condom** | □ | X |  |  |
| **Transparent membrane** | □ | X |  |  |
| **Sexual abstinence** | X |  |  |  |
| **Contraceptive plaster** | X |  |  |  |
| **Male pill** | X |  |  |  |
| **Ulipristal acetate** | X |  |  |  |
| **Cervical cap** | □ | X |  |  |
| **Vaginal washing** | □ |  | X |  |
| **Contraceptive sponge** | □ |  | X |  |

**78. Please indicate which of the following diseases can be transmitted by sex.**

| **DISEASE** | **Sexually transmitted** | **Non sexually transmitted** | **Do not know** |
| --- | --- | --- | --- |
| **Gonorrhea** | X |  |  |
| **Mononucleosis** | X |  |  |
| **Syphilis** | X |  |  |
| **AIDS** | X |  |  |
| **HPV Papilloma** | X |  |  |
| **Hepatitis C** | X |  |  |
| **Hepatitis B** | X |  |  |
| **Genital Herpes** | X |  |  |
| **Streptococcal infection** | **X** |  |  |
| **Hepatitis A** | **X** |  |  |
| **Venereal lymphogranuloma** | X |  |  |
| **Wart** | X |  |  |
| **Psoriasis** |  | **X** |  |
| **Candidiasis** | X |  |  |
| **Tuberculosis** | **X** |  |  |

**___________________________________ Thank you for your contribution to this research survey**
